# De novo SNP discovery and genetic linkage mapping in poplar using restriction site associated DNA and whole-genome sequencing technologies

**DOI:** 10.1186/s12864-016-3003-9

**Published:** 2016-08-18

**Authors:** Mohaddeseh Mousavi, Chunfa Tong, Fenxiang Liu, Shentong Tao, Jiyan Wu, Huogen Li, Jisen Shi

**Affiliations:** The Southern Modern Forestry Collaborative Innovation Center, College of Forestry, Nanjing Forestry University, Nanjing, 210037 China

**Keywords:** Restriction site associated DNA, Whole-genome sequencing, Single nucleotide polymorphism, Genetic linkage map, *Populus*

## Abstract

**Background:**

Restriction site associated DNA sequencing (RAD-seq), a next-generation sequencing technology, has greatly facilitated genetic linkage mapping studies in outbred species. RAD-seq is capable of discovering thousands of genetic markers for linkage mapping across many individuals, and can be applied in species with or without a reference genome. Although several analytical tools are available for RAD-seq data, alternative strategies are necessary for improving the marker quality and hence the genetic mapping accuracy.

**Results:**

We demonstrate a strategy for constructing dense genetic linkage maps in hybrid forest trees by combining RAD-seq and whole-genome sequencing technologies. We performed RAD-seq of 150 progeny and whole-genome sequencing of the two parents in an F1 hybrid population of *Populus deltoides* × *P. simonii*. Two rough references were assembled from the whole-genome sequencing reads of the two parents separately. Based on the parental reference sequences, 3442 high-quality single nucleotide polymorphisms (SNPs) were identified that segregate in the ratio of 1:1. The maternal linkage map of *P. deltoides* was constructed with 2012 SNPs, containing 19 linkage groups and spanning 4067.16 cM of the genome with an average distance of 2.04 cM between adjacent markers, while the male map of *P. simonii* consisted of 1430 SNPs and the same number of linkage groups with a total length of 4356.04 cM and an average interval distance of 3.09 cM. Collinearity between the parental linkage maps and the reference genome of *P. trichocarpa* was also investigated. Compared with the result on the basis of the existing reference genome, our strategy identified more high-quality SNPs and generated parental linkage groups that nicely match the karyotype of *Populus*.

**Conclusions:**

The strategy of simultaneously using RAD and whole-genome sequencing technologies can be applied to constructing high-density genetic maps in forest trees regardless of whether a reference genome exists. The two parental linkage maps constructed here provide more accurate genetic resources for unraveling quantitative trait loci and accelerating molecular breeding programs, as well as for comparative genomics in *Populus*.

**Electronic supplementary material:**

The online version of this article (doi:10.1186/s12864-016-3003-9) contains supplementary material, which is available to authorized users.

## Background

Forests cover about 30 % of Earth’s land area and are of significant economic and ecological importance [1]. Although most forest trees are characterized by their large and complex genomes, recent advances in DNA sequencing technology with assistances of available physical maps [2–4], have led to several tree genome sequence assemblies, which include two poplar species (*Populus trichocarpa* [5] and *P. euphratica* [6]), three conifer species (*Picea abies* [7], *Picea glauca* [8] and *Pinus taeda* [9]), and *Eucalyptus grandis* [10]. The availability of genome sequence information is essential for studying genomic architecture and evolution as well as for comparative genomics. However, for those tree species without a reference genome sequence, investigations on genome structure have to resort to genetic linkage maps that show the order and distance of a set of genome-wide genetic markers. Indeed, genetic linkage maps play an important role in genome comparisons with other species and assembling genome scaffold sequences or validating the integrity of an existing genome assembly [11–13]. More importantly, genetic linkage maps are prerequisites for identifying quantitative trait loci (QTLs) that control growth, wood quality, and other economically important traits, and thereby facilitating the genetic improvement of cultivated trees through marker-assisted selection and breeding.

Over the past two decades, more and more linkage maps have been constructed for a large number of forest trees [1, 14]. One of the major steps for constructing a linkage map is to obtain a set of genetic marker genotype data across many individuals in a mapping population [1]. Many previous tree linkage maps were established using molecular markers such as randomly amplified polymorphic DNA (RAPD), restriction fragment length polymorphisms (RFLP), amplified fragment length polymorphisms (AFLP), and simple sequence repeats (SSR). These traditional genetic markers were only developed to a small or moderate number due to instability or labor-intensive experiments, thus usually leading to sparse or unsaturated linkage maps, especially in outbred forest trees. Although recent single nucleotide polymorphism (SNP) array technologies have been applied to produce higher throughput marker data for constructing linkage maps in forest trees such as *Eucalyptus* [15] and *Populus* [16], they also have some limitations, including unreliable or useless genotype calls and only a small fraction of the studied loci being polymorphic [17].

Next-generation DNA sequencing (NGS) technologies can produce tremendous amounts of DNA sequence data at a consistently low cost, allowing us to obtain thousands of SNPs for genetic mapping. However, it is infeasible to apply whole-genome sequencing directly to hundreds of individuals in a mapping population because the total expense would be so high that most research projects cannot afford it. Therefore, several DNA library preparation methods for NGS have been developed to solve this problem by reducing genome complexity and adding DNA barcodes to samples [17]. These methods include restriction site associated DNA sequencing (RAD-seq) [18], genotyping-by-sequencing (GBS) [19], and specific locus amplified fragment sequencing (SLAF-seq) [20], and we focus on RAD-seq in the present study. RAD-seq uses NGS platforms for targeted sequencing of regions near restriction enzyme cut sites across genomes of many samples [18, 21]. RAD-seq has been extensively applied in constructing linkage maps in many organisms such as barley [22], ryegrass [23], moth [24], grape [25], gudgeon [26] and cotton [27]. Generally, there are two ways of discovering SNPs or other RAD markers, either with or without reference genome sequences. When a reference genome is available, RAD-seq reads can first be mapped to the reference sequences with tools such as BWA [28] and Bowtie2 [29], and then SNP calling or genotyping can be performed with tools such as SAMtools [30] and GATK [31]. For species without available reference genomes, de novo methods have to be employed to generate RAD markers, which include Stacks [32], RADtools [24], RaPiD [33], Rainbow [34] and PyRAD [21]. Although many tools are available for RAD-seq data analyses, there is still much room to improve the analytical strategies for obtaining more accurate and reliable SNP genotypes, particularly in highly heterozygous forest trees [35].

In this study, we performed RAD-seq data analysis for genetic mapping by combining the use of RAD-seq data from the progeny with the whole-genome sequencing data of their parents in an F1 hybrid population of *Populus deltoides* × *P. simonii*. The female parent *P. deltoides* has the characteristics of fast growth and resistance to disease but a poor rooting ability, while the male parent *P. simonii* has strong hardiness in cold, heat, drought and other bad conditions, and an excellent regeneration ability. The hybrids of the two parents display significant difference in morphological and physiological traits, providing a permanent material for mapping QTLs. Short paired-end (PE) reads of whole-genome sequencing data with high coverage from each parent were de novo assembled into contigs, forming a rough reference sequence of the parent. Based on each of the two parental reference sequences, two SNP datasets were identified and validated with each other to generate a high-quality SNP dataset for linkage mapping. Consequently, two high-quality parental linkage maps were constructed, each with a number of linkage groups that matched the karyotype of *Populus* perfectly. Collinearity between the parental linkage maps and the reference genome of *P. trichocarpa* was also investigated. Compared with the result of linkage mapping based solely on the reference genome of *Populus* [36], our strategy generated more accurate genetic linkage maps of the two parents. This strategy could be applied to construct high-density and high-quality genetic linkage maps, especially in outbred forest trees with or without a reference genome.

## Methods

### Plant materials and Illumina sequencing

The mapping material was a population of interspecific F1 hybrids between *P. deltoides* and *P. simonii*, which was generated in 2011 in Xiashu Forest Farm of Nanjing Forestry University, Jurong, Jiangsu Province, China [36]. We selected 150 individuals for genetic linkage mapping in this study. In the spring of 2013, young leaf tissue was collected and DNA was extracted from the two parents and 150 progeny using the CTAB protocol [37].

We performed RAD sequencing of the 150 progeny and whole-genome sequencing of the two parents. The RAD library for the progeny was constructed following the protocol described by Baird et al. [18] with a few modifications, details of which can be found in our previous study [36]. RAD sequencing was performed in seven lanes (PE, 90 bp) on an Illumina HiSeq 2000 at Beijing Genomics Institute (BGI), Shenzhen, China. For the whole-genome sequencing of the two parents, the DNA was randomly sheared by sonication and ligated with adapters. Fragments of 300–500 bp were selected using agarose gel electrophoresis. Two sequencing libraries for the two parents were constructed according to the Illumina protocol. The whole-genome sequencing was conducted on a HiSeq 2500 platform at Biomarker Technologies Co, Ltd. (BMK), Beijing, China.

The raw sequencing data were processed to clean the data with the same standard quality control pipelines in the two companies (i.e. BGI and BMK), and then to obtain high quality (HQ) data using NGS QC toolkit [38]. First, reads from each individual were segregated according to its unique molecular identifier. Second, paired reads containing primer/adapter sequence or having more than 10 % uncalled bases (N) were discarded. Third, paired reads were also discarded if more than 50 % of the bases in either of the reads have Phred quality score less than 5. And finally, we further filtered the clean data with NGS QC toolkit [38] to obtain HQ reads such that more than 70 % of the bases for each read have quality scores greater than or equal to 20.

### De novo assembly, SNP discovery and genotyping

To improve the quality of genome assembly, we used the Perl program ErrorCorrectReads.pl in ALLPATHS-LG [39] to correct base calling errors in the HQ reads from the two parents. Each parental genome was then assembled from its corrected short reads using SOAPdenovo [40], which builds contigs using a de Bruijn graph algorithm. Different *k*-mer lengths were used and the optimal assembly was selected according to several parameters such as N50 and average contig length.

The two sets of contigs assembled above were considered to be the rough genome sequences of the female *P. deltoides* ‘I-69’ and the male *P. simonii* ‘L-3’. We performed SNP calling and genotyping across the hybrid F1 population based on the two parental rough genome sequences and the reference genome sequence of *P. trichocarpa* separately [5], using the software BWA [28], SAMtools and BCFtools (v1.2, [30]), and several in-house Perl scripts with the following steps:

1. mapping the filtered HQ reads from each individual to a reference genome sequence to generate a sequence alignment/map (SAM) format file using the BWA *mem* command with default parameters;
2. filtering out those records having an edit distance greater than 9 or best alignment score less than 60 or second-best alignment score greater than the best alignment score in the SAM file of each individual;
3. converting the filtered SAM file to BAM format and then sorting and indexing with SAMtools;
4. producing BCF files with the command *samtools mpileup –g –I* for all individuals;
5. generating VCF files with the command *bcftools call –m –v* for each parent;
6. filtering SNPs from the parental VCF files such that each SNP has a mapping quality score of at least 20 and a read coverage depth (DP) of at least 5, and merging the two parental SNP datasets into a list site file;
7. for all individuals, including the two parents, creating VCF files with the command *bcftools call –m –f GQ –T* using the list site file generated in step (6);
8. calling genotypes at all the list sites for each individual and filtering using stringent conditions with DP ≥ 10 and genotyping quality (GQ) > 50;
9. generating a SNP genotype dataset for the common SNP sites across the two parents and all 150 progeny.

Finally, three SNP genotype datasets were generated on the basis of the genome sequences of *P. deltoides*, *P. simonii* and *P. trichocarpa*, denoted by PD, PS and PT, respectively. The SNPs in those datasets were further filtered for linkage mapping according to Mendel’s law of segregation.

### Linkage map construction

We performed chi-squared tests on all the SNPs in the PD, PS and PT datasets generated above to check whether they follow Mendel’s law of segregation. If a SNP deviated seriously from the Mendelian segregation ratio (*p* < .01) or had more than 10 % missing genotypes in the population, it was removed from linkage analysis. To use the SNPs called from the two parental rough genome sequences, the SNP loci that were identical between the filtered PD and PS datasets, at which each individual has the same genotype in the two datasets (i.e. the Hamming distance between PD and PS equal to 0 for a SNP locus), were chosen for linkage mapping. Because the overwhelming majority of SNPs were found to segregate in the ratio of 1:1 in the mapping population, we had to construct two parental linkage maps using the traditional pseudo-testcross mapping strategy [41] with the software packages JoinMap 4.1 [42] and FsLinkageMap 2.1 [43]. The maternal linkage map was constructed with the identical SNPs of segregation type *ab*×*aa*, and the paternal linkage map with the identical SNPs of segregation type *aa*×*ab*. For each linkage map construction, two-point linkage analysis was first performed for all pairs of SNP loci and then SNP markers were grouped under a logarithm of odds (LOD score) threshold using the software FsLinkageMap. Next, SNP markers in each linkage group were ordered three times using JoinMap with the maximum likelihood mapping method and once using FsLinkageMap. The optimal order was chosen as the mapping order of the linkage group according to the four ordering results with the two software packages and the ordering criterion of the minimum sum of adjacent recombination fractions [44]. Finally, map distances were calculated with the Kosambi mapping function, and linkage maps were first drawn in WMF format using FsLinkageMap and then modified in PDF or EPS format using the software Mayura Draw (http://www.mayura.com).

## Results

### Illumina sequencing and de novo assembly

We obtained 615,038,434 clean 101-bp reads from BMK, including 288,115,744 and 326,922,690 from whole genomes of the maternal *P. deltoides* ‘I-69’ and the paternal *P. simonii* ‘L-3’, respectively. RAD sequencing was performed at BGI, and 2,010,564,342 clean reads each 82–90 bp in length were generated with an average of 13,403,762 reads from each of the 150 progeny. We filtered out low-quality reads in which more than 30 % of bases had Phred quality score ≤ 20 using NGS QC toolkit for all individuals, and performed error correction only for each parental dataset with the standalone Perl script ErrorCorrectReads.pl in ALLPATHS-LG. This resulted in 231,587,056 (80 %) and 258,870,468 (79 %) high-quality reads for the parents ‘I-69’ and ‘L-3’, respectively, and an average of 13,001,952 (97 %) high-quality reads for the progeny. The final high-quality dataset of each parent was used for de novo assembly and each individual dataset for SNP genotype calling (Table 1).

**Table 1:** Summary of whole-genome sequencing and RAD-seq data from BMK and BGI with averages in brackets

Experiment	Sample	Number of sample	Number of clean reads	Clean reads data (Gb)	Number of high-quality reads	High-quality reads data (Gb)
BMK	Female parent	1	288,115,744	29.10	231,587,056	23.39
	Male parent	1	326,922,690	33.02	258,870,468	26.15
BGI	Progeny	150	2,010,564,342 (13,403,762)	137.37 (0.92)	1,950,292,874 (13,001,952)	123.23 (0.82)
Total	Total	152	2,625,602,776	199.49	2,440,750,398	172.77

*RAD-seq* restriction site associated DNA sequencing

The high-quality sequence reads of each parent were assembled using SOAPdenovo at different *k*-mer lengths of 21, 27, 31, 37, 41, 47, 51, 57, 61 and 67 with contig cut-off length of 150 bp. We considered the contig dataset of each assembly result and compared statistics such as total number of contigs, N50 length, average contig length and longest contig length. The *k*-mer length was generally proportional to the number of contigs, total length and longest contig length for the assemblies of each parent (Additional file 1). We chose the optimal assembly that had the highest N50 contig length and largest average contig length among the different *k*-mers. We found that the optimal assemblies of the two parents both corresponded to a *k*-mer length of 37, which resulted in N50 length of 586 bp and average contig length of 441 bp for the female ‘I-69’, and N50 length of 873 bp and average contig length of 532 bp for the male ‘L-3’. These optimal assemblies contained 767,393 and 664,721 contigs spanning the genome sizes of 338.39 and 353.79 Mb for the female ‘I-69’ and male ‘L-3’, respectively (Additional file 1). We used the two assemblies as rough reference genome sequences for SNP discovery and genotype calling across the whole hybrid family of *P. deltoides* × *P. simonii*.

### SNP discovery and genotype calling

We first mapped the high-quality reads of the two parents on the two rough reference genomes of *P. deltoides* and *P. simonii* and the reference genome of *P. trichocarpa* using the mapping tool BWA. As a result, more than 70 % of the high-quality reads from each parent were best mapped to their own rough reference sequence, while only 56–59 % were mapped to the other two reference sequences (Table 2). Each of these alignments had edit distance less than 9 and best alignment score at least 60 higher than that of the second-best alignment. With these mapping results in SAM format, we performed SNP calling using SAMtools and BCFtools. Based on the rough genome sequence of *P. deltoides*, 423,680 and 4,721,160 SNPs with coverage depth of at least 5 were discovered in the female parent ‘I-69’ and the male parent ‘L-3’, respectively. The number of SNPs found in both parents was 168,530, leading to 4,976,310 SNPs discovered in one or both parents. For convenience, we denote this total SNP dataset by PD. Similarly, we also found that the total numbers of SNPs in the two parents were 5,227,450 and 11,694,085 according to the reference sequences of *P. simonii* and *P. trichocarpa*, respectively (Table 2). These two SNP datasets are denoted by PS and PT.

**Table 2:** Percentage of high-quality reads best mapped to the three reference sequences and number of SNPs identified accordingly for the female parent ‘I-69’ and the male parent ‘L-3’

Parent	*P. deltoides*		*P. simonii*		*P. trichocarpa*	
	Mapped reads (%)	SNP	Mapped reads (%)	SNP	Mapped reads (%)	SNP
I-69	71.34	423,680	56.77	4,955,722	56.23	6,597,917
L-3	56.55	4,721,160	76.77	414,174	58.57	7,018,418
Both		168,530		142,446		1,933,395
Total		4,976,310		5,227,450		11,682,940

**Fig. 1:**
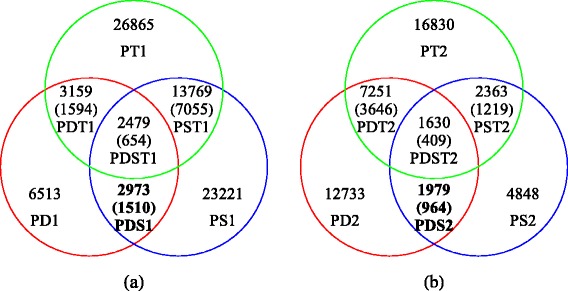
Venn diagram showing the numbers of identical SNPs between any two of or among three SNP data sets if the genotypes in the progeny are denoted as *aa* or *ab*. If the SNP genotypes are expressed using the nucleotide notations of A, C, G and T, the numbers are listed in brackets accordingly. (a) PD1, PS1, and PT1 are the SNP data sets calling from the two poplar parents based on the reference sequences of *P. deltoides*, *P. simonii* and *P. trichocarpa*, respectively, in which each SNP segregated in the Mendelian ratio of 1:1 with *p ≤* 0.01 and the segregation type of *ab*×*aa* and was genotyped in at least 90 % of the progeny. (b) PD2, PS2, and PT2 are the similar SNP data sets defined as in (a), but each SNP has the segregation type of *aa*×*ab*

Next, the high-quality reads from each progeny were also mapped to the three reference sequences separately and the best alignments were retained for SNP genotype calling. We performed SNP genotype calling across the whole population (two parents and 150 progeny) based on the three SNP datasets PD, PS and PT separately. After a series of filtering procedures (detailed in Materials and Methods), 6513 SNPs with segregation type of *ab*×*aa* were genotyped on the basis of SNP dataset PD, which followed the Mendelian segregation ratio of 1:1 with *p* ≥ .01 and at which at least 90 % of the progeny were genotyped. This new SNP dataset containing 6513 SNPs is denoted by PD1 for convenience. On the basis of SNP datasets PS and PT, the numbers of SNPs were 23,221 and 26,865, respectively, and these two new SNP datasets were accordingly denoted by PS1 and PT1. Further analyses revealed that 2973 SNPs in PD1 each corresponded to one or more SNPs in PS1, at which each progeny shared the same genotype if the genotype was denoted by *aa* or *ab*. We called these SNPs the identical SNP loci between the two SNP datasets PD1 and PS1 and denoted the set by PDS1. In the same way, the identical SNP datasets between PD1 and PT1, between PS1 and PT1, and among the three datasets PD1, PS1 and PT1 were denoted by PDT1, PST1, and PDST1, and their numbers of SNPs were 3159, 13,769 and 2479, respectively (Fig. 1(a)). If the genotype was denoted using the base symbols A, C, G and T, such as AA, AC, and GT, the numbers of identical SNPs between any pair or among all three datasets were abruptly reduced, as shown in brackets in Fig. 1(a).

For those SNPs having the segregation type *aa*×*ab*, following the Mendelian segregation ratio of 1:1 with *p* ≥ .01 and genotyped in at least 90 % of the progeny, the datasets generated from PD, PS and PT were denoted by PD2, PS2 and PT2, respectively. Also, the identical SNP datasets between PD2 and PS2, between PD2 and PT2, between PS2 and PT2, and among the three datasets PD2, PS2 and PT2 were denoted by PDS2, PDT2, PST2, and PDST2, respectively. The numbers of SNPs contained in these datasets are shown in Fig. 1(b).

### Genetic linkage maps

**Fig. 2:**
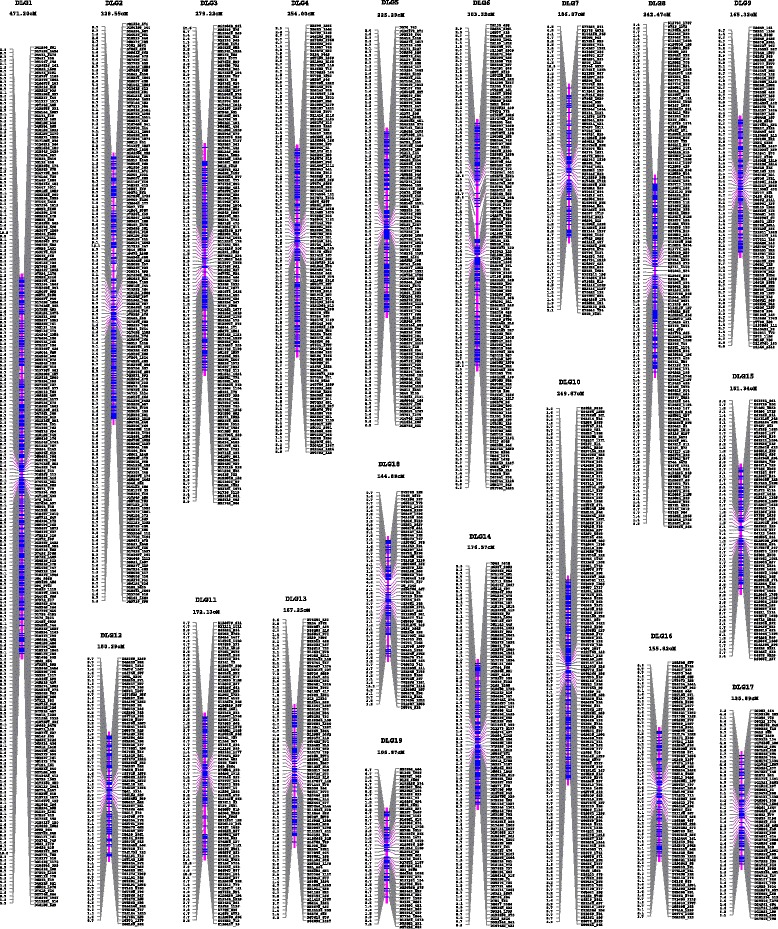
The genetic map of linkage groups DLG1-DLG19 for the maternal *P. deltoides* ‘I-69’. The length of each linkage group is presented under the linkage group name. Each SNP is named by the contig number of the rough reference sequence of *P. deltoides* and its position on it, prefixed with letter D

**Fig. 3:**
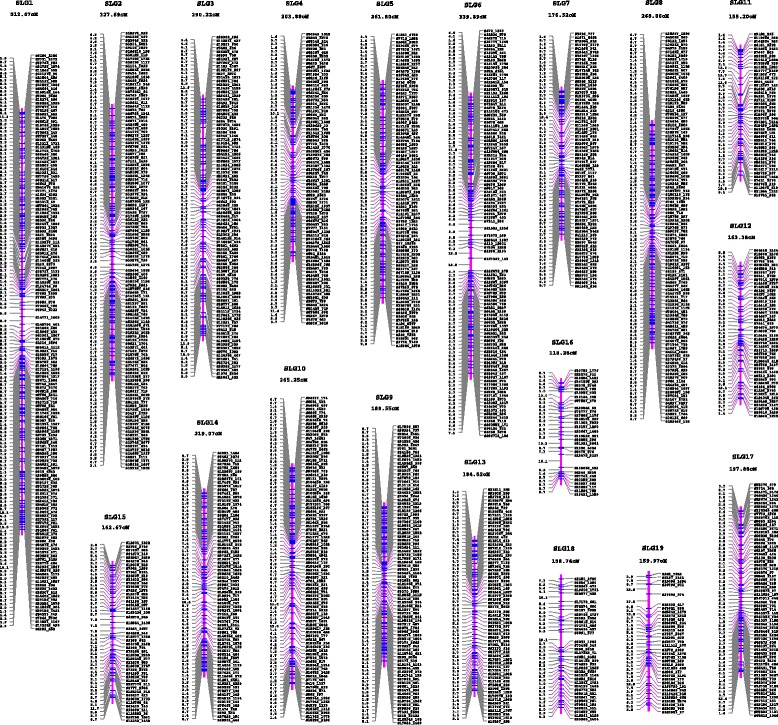
The genetic map of linkage groups SLG1-SLG19 for the paternal *P. simonii* ‘L-3’. The length of each linkage group is presented under the linkage group name. Each SNP is named by the contig number of the rough reference sequence of *P. simonii* and its position on it, prefixed with letter S

We used the two SNP datasets PDS1 and PDS2 generated above to construct genetic linkage maps of the maternal *P. deltoides* ‘I-69’ and the paternal *P. simonii* ‘L-3’, respectively. To improve mapping efficiency, when two or more SNPs were identical (*i.e.* complete linkage) within PDS1 or PDS2, and adjacent SNPs were within 1 kb on a contig, a single SNP was chosen to represent the group for linkage analysis. This reduced the number of SNPs in PDS1 from 2973 to 2012 and in PDS2 from 1979 to 1430. The final 2012 SNPs with segregation type *ab*×*aa* were assigned to 19 linkage groups (denoted as DLG1-19, Fig. 2), nicely matching the karyotype of *Populus*, at high LOD thresholds ranging from 6 to 18. After SNPs within each linkage group were ordered, a genetic linkage map of the female parent ‘I-69’ was constructed, spanning 4067.16 cM in total length with the individual groups ranging from 106.87 to 471.20 cM (Table 3). The distance between adjacent SNP markers on this genetic map ranged from 0.67 to 21.68 cM with an average of 2.04 cM (±1.69 SD). In the same way, the final 1430 SNPs with segregation type *aa*×*ab* were grouped into 19 linkage groups (denoted as SLG1-19, Fig. 3) at LOD thresholds ranging from 6 to 14, and they constituted a genetic linkage map of the male parent ‘L-3’ when SNPs within each linkage group were ordered. The total length of this paternal linkage map was 4356.04 cM, with the linkage group lengths varying from 118.28 to 512.67 cM, and the adjacent SNP intervals ranged from 0.67 to 19.05 cM with an average length of 3.09 cM (±2.41 SD) (Table 3). More detailed information on the two linkage maps is presented in Additional files 2 and 3, including SNP interval distance, cumulative distance, predicted linkage phase between adjacent SNPs, and the corresponding identical SNPs identified on the basis of the *P. trichocarpa* reference genome.

**Table 3:** SNP number and length of linkage groups in the two parental genetic maps of *P. deltoides* ‘I-69’ and *P. simonii* ‘L-3’

*P. deltoides* ‘I-69’			*P. simonii* ‘L-3’			Chromosome size (Mb)^a^
Group	SNP number	Length (cM)	Group	SNP number	Length (cM)	
DLG1	240 (196)^b^	471.20	SLG1	155 (121)	512.67	50.50
DLG2	162 (133)	328.55	SLG2	119 (98)	327.69	25.26
DLG3	134 (113)	279.22	SLG3	94 (74)	290.22	21.82
DLG4	120 (87)	254.00	SLG4	80 (60)	203.88	24.27
DLG5	112 (92)	225.29	SLG5	86 (71)	261.80	25.89
DLG6	130 (109)	303.32	SLG6	106 (88)	339.89	27.91
DLG7	81 (69)	186.87	SLG7	70 (57)	176.52	15.61
DLG8	141 (125)	242.47	SLG8	107 (88)	268.86	19.47
DLG9	90 (77)	165.32	SLG9	82 (68)	189.55	12.95
DLG10	145 (119)	249.87	SLG10	89 (75)	265.25	22.58
DLG11	85 (70)	172.13	SLG11	45 (32)	155.20	18.50
DLG12	75 (61)	150.29	SLG12	46 (37)	163.38	15.76
DLG13	85 (71)	167.25	SLG13	64 (60)	184.52	16.32
DLG14	102 (83)	176.57	SLG14	74 (60)	219.07	18.92
DLG15	73 (62)	151.34	SLG15	48 (44)	162.67	15.28
DLG16	72 (61)	155.82	SLG16	30 (24)	118.28	14.49
DLG17	59 (47)	135.89	SLG17	64 (49)	197.88	16.08
DLG18	61 (47)	144.89	SLG18	36 (29)	158.74	16.96
DLG19	45 (32)	106.87	SLG19	35 (22)	159.97	15.94
Total	2012 (1654)	4067.16		1430 (1157)	4356.04	394.51

^a^The genome size of *P. trichocarpa* (Tuskan et al. 2006)

^b^The number (in brackets) of SNPs identical to the SNPs calling based on the reference genome of *P. trichocarpa*

Further analyses revealed that there remained strong positive correlations between SNP number within a linkage group, linkage group length, and the physical size on the *P. trichocarpa* reference genome (Table 4). SNP number was highly correlated with linkage group length, with a correlation coefficient of 0.9731 for the maternal linkage map and 0.9406 for the paternal linkage map. The SNP number and length of the maternal linkage groups were also highly correlated with those of the paternal map with correlation coefficients more than 0.90. Moreover, the correlations between the linkage group lengths for the two parental maps and the physical size on the reference genome were also high with the coefficients of about 0.92. However, the correlations between the SNP numbers of linkage groups for the two parental maps and the physical size were relatively lower, but still having high coefficients over 0.80.

**Table 4:** Correlations among the SNP number, genetic length and chromosome size for the linkage groups of the two parental maps

	SNP number in DLG	DLG Length	SNP number in SLG	SLG length
DLG Length	0.9731			
SNP number in SLG	0.9334	0.9199		
SLG length	0.9338	0.9495	0.9406	
Chromosome size	0.8852	0.9202	0.8018	0.9231

In spite of the high positive correlations described above, we observed that there existed some unusual patterns between the two parental linkage groups either in length or in the number of SNPs. When we compared the length of each male linkage group with the corresponding female linkage group, we found that the difference of >3 cM per 1 Mb occurred in two linkage groups, i.e. LG17 (3.86 cM) and LG19 (3.33 cM). Interestingly, it was noted that DLG19 has more number of SNPs (45) than SLG19 (35) while its length (106.87 cM) is significantly shorter than that of the later (159.97 cM). This could be explained by the recombination suppression phenomenon possibly occurred on chromosome 19 in the female parent due to the sex determination through a ZW system in *Populus* [45]. For the same reason, we inferred that recombination could be also suppressed on chromosome 17 in the maternal parent *P. deltoides* ‘I-69’.

### Collinearity between genetic and physical maps

**Fig. 4:**
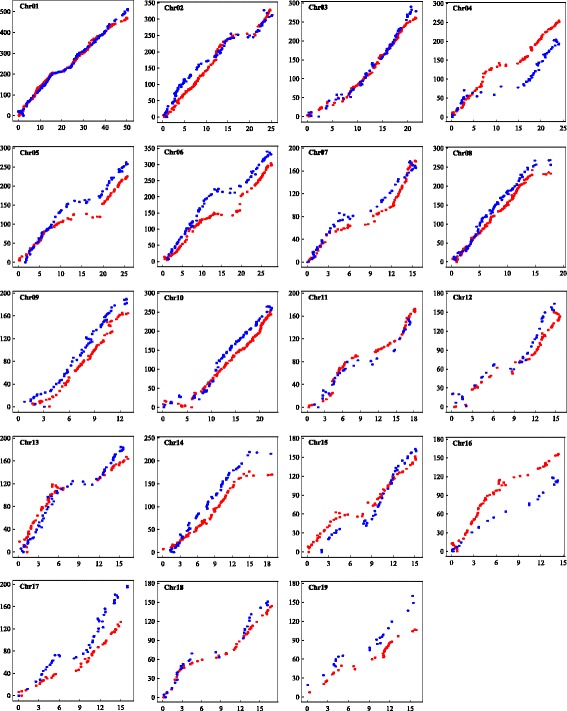
Collinear comparison between the parental genetic maps and the reference genome of *P. trichocarpa*. The x-axis indicates the reference sequence position with the unit of Mb; the y-axis indicates the genetic map position with the unit of cM. The red and blue points, respectively, indicate the SNP positions on the maternal and paternal genetic maps against the identical SNP positions on the reference genome

We found that 1654 (82.2 %) SNPs on the maternal linkage map and 1157 (80.9 %) SNPs on the paternal linkage map segregated identically to at least one SNP in the PT1 or PT2 dataset, in which each SNP has its position information on the reference genome of *P. trichocarpa* (Additional files 2 and 3). These identical SNPs connected the linkage groups of the two parents to the chromosomes of the reference genome, allowing direct comparisons between the genomes of *P. deltoides*, *P. simonii* and *P. trichocarpa*. Figure 4 presents scatter plots of the genetic map positions of the identical SNPs against their physical positions on the reference genome of *P. trichocarpa* for the 19 linkage groups of the two parents. On the whole, there is apparently a high level of collinearity between the parental linkage groups and the chromosomes of the reference genome. However, almost all of the linkage groups showed one or more local regions where SNP orders were inconsistent with the reference genome positions.

## Discussion

Here we demonstrated a novel strategy for constructing high-quality genetic linkage maps in forest trees by combining the use of RAD-seq with whole-genome resequencing technologies. This genetic mapping strategy may be applicable to most outbred forest species in which no reference genome is available. With the plummeting cost of NGS, it is feasible for most laboratories to perform RAD-seq across tens to hundreds of individuals and to sequence the whole genomes of their two parents in an F1 hybrid population. Assemblies from the parental whole-genome sequencing reads can be used as rough references, and RAD-seq reads of each progeny as well as the reads from each parent can be aligned to them separately. Thus, hundreds of thousands of SNPs could be discovered and genotyped across the population with available software packages designed for NGS data, and then thousands of high-quality SNP markers may be selected for genetic linkage mapping, in terms of the Mendelian segregation ratio, the fraction of missing genotypes and other features such as mapping quality and read coverage depth. Two SNP genotype datasets are generally derived from the two parental rough reference genomes and can validate each other using the Hamming distance across the individuals to improve mapping data quality substantially. After obtaining such a large number of high-quality SNP genotypes across the F1 mapping population, current linkage mapping tools are applied with the strategy of choosing the best marker orders (as described in Materials and Methods) to construct parent-specific dense linkage maps.

RAD-seq has been extensively applied to SNP and RAD marker discovery across populations in species with or without a reference sequence [18, 35]. When a reference genome is unavailable, de novo methods can be used with several tools such as Stacks [32] and RADtools [24]. However, few comparison studies have been carried out to evaluate the performance of different RAD-seq analytical strategies, including assembly and SNP calling software packages. Here we have presented an alternative method for calling SNPs across the F1 population using RAD-seq data by incorporating the whole-genome sequencing data of the two parents. Although a reference sequence is available for poplar ([5], https://phytozome.jgi.doe.gov/pz/portal.html), the assembled rough reference sequences of the two parents, *P. deltoides* and *P. simonii*, may be more appropriate for SNP and genotype calling in our mapping population, because there are divergences between the *P. trichocarpa* reference genome and the two parental genomes. Furthermore, the reference genome assembly is not perfect with, to date, more than one thousand scaffolds still unassigned to any chromosomes. In a previous study [36], we mapped RAD-seq data from the same mapping population directly to the reference sequence of *P. trichocarpa* for SNP marker discovery and obtained 20 linkage groups for each parental linkage map, each with one linkage group ambiguously assigned to any chromosome. In contrast, in this study, we used the parental rough reference sequences and RAD-seq data from only half of the progeny to generate 19 linkage groups in each parental linkage map, which perfectly matches the karyotype of *Populus* (2n = 38).

Like most genetic mapping studies in forest trees [11, 46, 47], we obtained two parent-specific linkage maps using two SNP datasets generated from RAD-seq technology. The results of two sex-specific linkage maps from RAD-seq data can be found in other organisms such as ryegrass [23] and grape [25]. The main reason is that, for two diverged parents, at the overwhelming majority of SNP sites one parent has a heterozygous genotype and the other a homozygous genotype. Because a pair of SNPs, one with segregation type of *aa*×*ab* and the other *ab*×*aa*, cannot provide any recombination information [48], the two high-quality SNP datasets PDS1 and PDS2 generated in this study have to be analyzed separately, leading to two parental linkage maps. To construct an integrated genetic linkage map for the F1 hybrid population, a sufficient number of fully informative SNP markers with segregation type of *ab*×*ab* or *ab*×*cd* should be identified as bridges to link the two types of SNPs segregating in 1:1 ratio. With the decreasing cost of NGS, this could be resolved by resequencing the whole genomes instead of RAD-seq across many individuals in the mapping population to identify more fully informative SNPs.

We used 150 progeny for calling genotypes from RAD-seq data and for the subsequent construction of the genetic linkage maps. Such a moderate sample size can provide enough information to estimate the recombination fraction accurately between any two genetic markers that follow a Mendelian segregation ratio of 1:1. Because there are only four combined genotypes for a pair of markers each with a segregation ratio of 1:1, the average count of the combined genotypes was about 38 in our mapping population, which led to an expected LOD score of 5.36 for two moderately linked loci with a recombination fraction of 0.30 [49, 50]. This indicates that such a sample size could allow a large number of the moderately and tightly linked markers contained in a genetic linkage map, with a maximal genetic distance of 34.66 cM between two adjacent markers under the Kosambi mapping function. In summary, a moderate sample size of about 150 or more individuals is recommended for constructing parent-specific genetic linkage maps in an F1 hybrid population of forest trees with RAD-seq and whole-genome sequencing technologies.

## Conclusions

Assembled contigs of whole-genome PE reads from each parent in an F1 hybrid population can be used as a rough reference for performing SNP calling and genotyping with RAD-seq data across the whole population. The two SNP genotype datasets each based on one parental reference can confirm each other to generate a high-quality genotype dataset for linkage mapping. This strategy could be applied to highly heterozygous undomesticated forest trees with or without a reference genome to construct high-density genetic linkage maps, which is difficult with traditional molecular markers. Both of the parental genetic linkage maps of *P. deltoides* and *P. simonii* constructed here with high density and quality perfectly match the karyotype of *Populus*, and provide important genetic resources for identifying QTLs, accelerating molecular breeding programs and performing comparative genomics in *Populus*.

## Additional files

Additional file 1: De novo assembly statistics of *P. deltoides* ‘I-69’ and *P. simonii* ‘L-3’ with different *k*-mers. (DOCX 20 kb) Additional file 2: Detailed information on genetic distance and linkage phase between adjacent SNP markers on the genetic linkage map of the female *P. deltoides* ‘I-69’. The corresponding identical SNPs identified based on the *P. trichocarpa* reference genome are also included. (XLS 452 kb) Additional file 3: Detailed information on genetic distance and linkage phase between adjacent SNP markers on the genetic linkage map of the male *P. simonii* ‘L-3’. The corresponding identical SNPs identified on the basis of the *P. trichocarpa* reference genome are also included. (XLS 349 kb)

## Abbreviations

BGI: Beijing Genomics Institute
BMK: Beijing’s Biomarker Technologies Co, Ltd
DP: coverage depth
GQ: genotyping quality
HQ: high quality
NGS: next-generation DNA sequencing
PE: paired-end
QTL: quantitative trait loci
RAD-seq: restriction site associated DNA sequencing
SAM: sequence alignment/map
SNP: single nucleotide polymorphism
