# Supplementary material for: De novo SNP discovery and genetic linkage mapping in poplar using restriction site associated DNA and whole-genome sequencing technologies
Source: BMC Genomics. 2016 Aug 18;17:656. doi: 10.1186/s12864-016-3003-9 (PMC4991039; doi:10.1186/s12864-016-3003-9)
Supplement: Additional file 1: — De novo assembly statistics of P. deltoides ‘I-69’ and P. simonii ‘L-3’ with different k-mers. (DOCX 20 kb) [file 12864_2016_3003_MOESM1_ESM.docx]

***De novo* assembly statistics of the *P. deltoides* ‘I-69’ and *P. simonii* ‘L-3’ with different *k*-mers.**

| Parent | *K*-mer length | Number of  contigs | Total length (Mb) | Contig N50 (bp) | Average contig length (bp) | Logest contig (bp) |
| --- | --- | --- | --- | --- | --- | --- |
| *P. deltodes* | 21 | 562168 | 219.06 | 474 | 389 | 14256 |
|  | 27 | 655688 | 283.35 | 559 | 432 | 19071 |
|  | 31 | 701062 | 308.21 | 578 | 440 | 20922 |
|  | 37 | 767393 | 338.39 | 586 | 441 | 20928 |
|  | 41 | 812774 | 356.32 | 584 | 438 | 20928 |
|  | 47 | 879328 | 381.16 | 577 | 433 | 19928 |
|  | 51 | 916772 | 395.19 | 569 | 431 | 19932 |
|  | 57 | 939827 | 408.49 | 563 | 435 | 23235 |
|  | 61 | 944805 | 414.05 | 562 | 438 | 23235 |
|  | 67 | 930511 | 414.86 | 563 | 446 | 20106 |
|  |  |  |  |  |  |  |
| *P. simonii* | 21 | 535035 | 229.99 | 563 | 430 | 9942 |
|  | 27 | 584397 | 298.11 | 771 | 510 | 19516 |
|  | 31 | 613366 | 323.51 | 833 | 527 | 23080 |
|  | 37 | 664721 | 353.79 | 873 | 532 | 33794 |
|  | 41 | 703823 | 371.48 | 876 | 527 | 33798 |
|  | 47 | 763299 | 395.93 | 860 | 519 | 34616 |
|  | 51 | 800760 | 410.24 | 839 | 512 | 34676 |
|  | 57 | 831585 | 425.27 | 811 | 511 | 34688 |
|  | 61 | 839506 | 431.66 | 797 | 514 | 34089 |
|  | 67 | 825750 | 432.99 | 789 | 524 | 32960 |
